# Somatostatin receptor subtype expression and radiomics from DWI-MRI represent SUV of [68Ga]Ga-DOTATOC PET in patients with meningioma

**DOI:** 10.1007/s11060-023-04414-3

**Published:** 2023-09-14

**Authors:** Sarah Iglseder, Anna Iglseder, Vincent Beliveau, Johanna Heugenhauser, Elke R. Gizewski, Johannes Kerschbaumer, Guenther Stockhammer, Christian Uprimny, Irene Virgolini, Jozsef Dudas, Meinhard Nevinny-Stickel, Martha Nowosielski, Christoph Scherfler

**Affiliations:** 1 Department of Neurology, Innsbruck Medical University, Innsbruck, Austria; 2 Department of Geodesy and Geoinformation, Technical University Vienna, Vienna, Austria; 3 Neuroimaging Research Core Facility, Innsbruck Medical University, Innsbruck, Austria; 4 Department of Neuroradiology, Innsbruck Medical University, Innsbruck, Austria; 5 Department of Neurosurgery, Innsbruck Medical University, Innsbruck, Austria; 6 Department of Nuclear Medicine, Innsbruck Medical University, Innsbruck, Austria; 7 Department of Otorhinolaryngology, Innsbruck Medical University, Innsbruck, Austria; 8 Department of Therapeutic Radiology and Oncology, Innsbruck Medical University, Innsbruck, Austria

**Keywords:** ADC maps, Radiomic features, SUV_max_, [68Ga]Ga-DOTATOC PET, Meningioma

## Abstract

**Objective:**

This retrospective study aimed to analyse the correlation between somatostatin receptor subtypes (SSTR 1–5) and maximum standardized uptake value (SUV_max_) in meningioma patients using Gallium-68 DOTA-D-Phe1-Tyr3-octreotide Positron Emission Tomography ([68Ga]Ga-DOTATOC PET). Secondly, we developed a radiomic model based on apparent diffusion coefficient (ADC) maps derived from diffusion weighted magnetic resonance images (DWI MRI) to reproduce SUV_max_.

**Method:**

The study included 51 patients who underwent MRI and [68Ga]Ga-DOTATOC PET before meningioma surgery. SUV_max_ values were quantified from PET images and tumour areas were segmented on post-contrast T1-weighted MRI and mapped to ADC maps. A total of 1940 radiomic features were extracted from the tumour area on each ADC map. A random forest regression model was trained to predict SUV_max_ and the model’s performance was evaluated using repeated nested cross-validation. The expression of SSTR subtypes was quantified in 18 surgical specimens and compared to SUV_max_ values.

**Results:**

The random forest regression model successfully predicted SUV_max_ values with a significant correlation observed in all 100 repeats (p < 0.05). The mean Pearson’s r was 0.42 ± 0.07 SD, and the root mean square error (RMSE) was 28.46 ± 0.16. SSTR subtypes 2A, 2B, and 5 showed significant correlations with SUV_max_ values (p < 0.001, R2 = 0.669; p = 0.001, R2 = 0.393; and p = 0.012, R2 = 0.235, respectively).

**Conclusion:**

SSTR subtypes 2A, 2B, and 5 correlated significantly with SUV_max_ in meningioma patients. The developed radiomic model based on ADC maps effectively reproduces SUV_max_ using [68Ga]Ga-DOTATOC PET.

**Supplementary Information:**

The online version contains supplementary material available at 10.1007/s11060-023-04414-3.

## Introduction

Magnetic resonance imaging (MRI) is the gold standard to diagnose meningioma and represents an important imaging tool for surgical as well as radiation treatment planning, monitoring and follow-up after treatment [1]. Following meningioma surgery, conventional neuroimaging with MRI has limitations in distinguishing between tumour remnants and adjacent anatomical structures, postoperative changes (e.g., scars) [2] and/or bone involvement [3]. This is particularly important for subsequent treatment planning such as (re-)resection or radiation therapy (i.e., definition of the target volume). A further challenge in meningioma management is the early prediction of tumour recurrence or progression. Studies have shown that positron emission tomography (PET) imaging can overcome some of these challenges.

Somatostatin receptors (SSTR) are one of the main targets for PET imaging of meningiomas. High levels of SSTR subtype 2 expression were found in meningioma compared to a very low expression in adjacent structures like brain tissue or bone [4–6]. Gallium-68 [68Ga]Ga–labeled SSTR ligands (DOTATOC, DOTATATE, DOTANOC) with high affinity to these receptors have therefore been shown to add valuable diagnostic information during meningioma management [2, 7–9]. [68Ga]Ga-DOTATOC PET has the ability to differentiate between tumorous and non-tumorous areas in regions with low MRI contrast [2, 8]. Due to the good tumour/non-tumour contrast, [68Ga]Ga-DOTATOC PET has also been used for radiation planning [10–13] with the goal to spare as much critical tissue as possible without missing tumour. It was also shown that [68Ga]Ga-DOTATOC PET maximum standardized uptake value (SUV_max_) predicted faster growth in World Health Organization (WHO) grades I and II meningioma [9]. To determine tracer uptake intensity in PET imaging, SUV_max_ is used to supplement visual interpretation and it represents the tissue radioactivity concentration [14]. A correlation between SSTR2 expression and corresponding SUV_max_ was found [15] in neuronavigated tissue biopsies. No correlation of SSTR subtypes (especially 2A und 2B) with SUV_max_ from [68Ga]Ga-DOTATOC PET has been done so far in meningioma patients. Although, SSTR-directed PET provides additional diagnostic information, it is not routinely integrated into the first-line diagnostic evaluation of meningiomas as not every neuro-oncologic center has the availability of a PET scanner. Hence, obtaining maximum information from MRI images which are acquired in clinical routine, is desirable.

Diffusion-weighted imaging (DWI) is a broadly available MRI sequence used to provide quantitative information on the diffusion of water molecules within the brain tissue and is an integral part of standard brain tumour imaging [16]. Radiomics is a method introduced to characterize complex structural properties from imaging data such as texture, shape, or decencies among neighbouring voxels. Radiomics has been shown to have numerous applications in neuroradiology [17] and could help the assessment of tumour phenotypes from routine medical images by providing additional quantitative information. Indeed, several studies investigating radiomics features derived from DWI MRI in meningioma patients already exist [18].

In this study, we aim to investigate the pathophysiological background of the SUV_max_ signal by comparing it to the expression of SSTR subtypes in meningioma tissue. As DWI MRI and ADC maps have been associated to information on cellular density [19] and properties of the extracellular matrix [20, 21] we hypothesize that the complex information described by radiomic features may contain signal related to [68Ga]Ga-DOTATOC PET/CT SUV_max_ values. For this purpose, we trained and evaluated a predictive model for inferring SUV_max_ values from radiomic features of the meningioma derived from ADC maps.

## Materials and methods

### Patient data and imaging

The protocol for our retrospective study was reviewed and approved by the local independent ethics committee (UN5202, 328/4.16). From January 2006 to February 2020, all patients who underwent a cranial MRI and [68Ga]Ga-DOTATOC PET/CT prior to surgery for cranial meningioma (first diagnosis and recurrent tumour) were evaluated. All meningiomas included in this study were surgically resected and the histologic analysis was done according the WHO criteria 2006 and 2016 (depending on the time point of study inclusion of the patients) [22, 23]. The inclusion criteria for the imaging part of the study were as follows: (1) histologically confirmed meningioma, (2) preoperative MRI (including T1 sequences with and without contrast enhancement, T2/FLAIR sequences, DWI-MRI with ADC maps) and [68Ga]Ga-DOTATOC PET/CT images within a time interval of 180 days, (3) determination of extent of surgical resection (Simpson Grade) by reviewing of surgical documentations in combination with pre- and postoperative MRI findings. The exclusion criteria were as follows: (1) ADC maps with other b-values than 0 and 1000 s/mm^2^, (2) incomplete or severe artefacts in MRI or PET images/sequences, (3) in case of recurrent meningioma, other interventions (e.g. chemotherapy, radiation therapy) except for prior surgery. In case of multiple meningiomas, the resected meningioma was used for analysis. Immunohistochemical analysis was performed in patients with sufficient and, above all, good tissue quality for immunohistochemical staining.

After consideration of the inclusion and exclusion criteria, 51 consecutive patients were included in this study with 35 low grade (WHO I) and 16 high grade (WHO II and III) meningiomas. Twelve (23,5%) patients had recurrent tumour. Importantly, these recurrent tumours were treatment naive, hence no other therapy (i.e., radiation) except for surgery has been applied before. See Table 1 for further patient details.

**Table 1:** Patients’ and meningioma characteristics

Patients	51
male : female (ratio)	21 : 30 (1 : 1,4)
median age at resection (range)	54 years (21 – 85 years)
multifocal meningioma	12 (23,5%)
Meningiomas	51
World Health Organization (WHO) grade	
I	35 (68,6%)
II	11 (21,6%)
III	5 (9,8%)
primary : recurrent	39 (76,5%) : 12 (23,5%)
Histological classification of meningiomas	
Meningothelial	22 (43,1%)
Fibroblastic	2 (3,9%)
Microcystic	1 (2,0%)
Secretory	3 (5,9%)
Transitional	7 (13,7%)
Atypical	11 (21,6%)
Anaplastic	5 (9,8%)
Tumor site	
Olfactorius nerve	1 (2,0%)
Sphenoidal	11 (21,6%)
Petroclival/clival	3 (5,8%)
Frontoparietal/-basal	7 (13,7%)
Sphenoorbital	4 (7,8%)
Parasagittal/falx	13 25,5%)
Infratentorial	1 (2,0%)
Convexity	11 (21,6%)
Extent of Resection (Simpson grading for meningioma)	
Gross-total resection (Simpson grade I-III)	39 (76,5%)
Subtotal resection (Simpson grade IV-V)	12 (23,5%)

### MRI and PET data acquisition

Patients have been investigated on different MRI scanners using 3 Tesla (T) and 1,5T (Siemens Symphony Vision (n = 5), Siemens Symphony Tim (n = 26), Siemens Avanto (n = 1), Siemens Sonata (n = 1), Siemens Aera (n = 5), Siemens Skyra (n = 9), GE Optima (n = 1) and Philips Achieva (n = 3)). Importantly, diffusion weighting was applied with b-values at 0 and 1000 s/mm^2^ in all patients. For details on the imaging protocol, please see **Supplement** Table 1. [68Ga]Ga-DOTATOC PET/CT scans were performed at the Department of Nuclear Medicine at Innsbruck Medical University using a dedicated PET/CT system General Electric (GE Discovery 690).[68Ga]Ga was obtained from a [68Ge]/[68Ga] radionuclide generator (Eckert & Ziegler, Berlin; Germany). The described method by Decristoforo et al. [24] was used for synthesis of [68Ga]Ga-DOTATOC and it was applied intravenously followed by a tracer uptake phase of 60 min. A contrast enhanced low-dose CT scan (Siemens Medical Solutions, Erlangen, Germany) of the head was performed for attenuation correction. The PET scan was acquired in a single bed position and the duration of acquisition was 5 min in emission mode, starting 60 min after application. PET emission data were reconstructed as axial, coronal and sagittal [25].

### MRI processing

Individual 3D T1 weighted MR images were segmented into gray matter, white matter and cerebrospinal fluid (CSF) compartments using statistical parametric mapping (SPM, Wellcome Department of Cognitive Neurology, London, United Kingdom). To compensate for eddy currents, DWI images were registered to an individual reference image without diffusion weighting (3D T1) using SPM [26]. Registered DWI were visually verified for correct calculation and reconstruction for every subject. Individual T1 post contrast enhanced images were used to segment the contrast enhancing tumour region and generate volumes of interest (VOIs). T2 weighted images were used to segment the T2 hyperintense voxels surrounding the tumour (edema). A semiautomatic segmentation method based on a signal intensity threshold and margin-based algorithms (ITK-SNAP 3.8.0) was used for this segmentation. This approach was previously shown to have high efficiency and produce reliable 3D segmentations [27]. Necrotic tissue was excluded from the segmentation. The manual labelling was performed by one experienced investigator (SI) in image segmentation with 8 years of experience.

In order to standardize ADC values among MRI scanners, previously delineated areas of the tumour and edema as well as the compartment of the CSF were deduced from the gray and white matter compartments. Consecutively, the ratio of ADC values of each individual voxel within the individual compartments (tumour, edema and the tumour-free compartment) was calculated. In order to avoid contamination from CSF and non-brain compartments due to partial volume effects, ADC voxel values that were outside a threshold of mean CSF ADC of 2 SD (standard deviations), determined for each individual, were excluded.

### Radiomic features

Radiomic features were extracted from the ADC maps within the manually defined VOI using PyRadiomics v3.0.1 [28]. PyRadiomics implements 8 pre-processing filters and 7 classes of radiomic feature leading to a total of 1940 unique radiomic features. The radiomic features included first order features, shape features (3D and 2D), gray level co-occurrence matrix (GLCM) features, gray level size zone matrix (GLSZM) features, gray level run length matrix (GLRLM) features, neighbouring gray tone difference matrix (NGTDM) features and gray level dependence matrix (GLDM) features. Detailed descriptions of the pre-processing filters and radiomic features can be found in the online documentation (https://pyradiomics.readthedocs.io).

### Analysis of [68Ga]Ga-DOTATOC PET/CT imaging

The PET/CT images were interpreted visually and semi-quantitatively by an experienced nuclear medicine physician (CU, 10 years of experience). Regions of interest (ROIs) were drawn manually around the hypermetabolic tumour lesions by a nuclear medicine physician on a Hermes Workstation (Hermes Medical Solutions, Stockholm, Sweden). To discriminate between tumour and non-tumoural tissue, we utilized the established SUV_max_ threshold of 2.3, as determined by Rachinger et al. [8]. The ROIs were adjusted in 3 planes so that the entire meningioma was included. SUV_max_ within the ROI was calculated by determining the maximum PET tracer uptake and correlating it with the applied dose and patients body weight. The highest SUV_max_ was recorded and used for further analysis.

### Statistical analysis, feature selection and model construction

Within the statistical data analysis, the radiomics derived from the ADC maps are further used as predictor variables to establish a model for the corresponding SUV_max_ values. Therefore, a random forest regression model (ranger 0.14.1) [29] was used. The model was evaluated using a repeated nested cross-validation (CV) design with 100 repeats and 10 and 5 folds for the outer and nested loops, respectively. To reduce the correlation between the features, optimize performance and avoid overfitting, a two-step feature selection was performed within each fold. Firstly, the number of features was narrowed down from 1940 to 30 using minimum redundancy maximum relevance (mRMR) feature selection [30]. Secondly, the number of features was further reduced using recursive feature elimination (rfe) [31]. Following this, the hyperparameters of the random forest regression models (mtry, split rule and minimum node size) were tuned to optimize the model. Details can be found in the online documentations of the according software packages mRMRe (2.1.2) [32] and caret (6.0–93) as well as feature selection and model tuning [33]. Default settings were used. The final model was applied on the test data within each fold. The cross-validation was repeated 100 times to obtain a realistic distribution of the prediction accuracy. This resulted in 100 predicted SUV_max_ values for each of the 51 subjects. For each repeat, the predicted and the observed values were compared by calculating the root mean square error (RMSE) and a Pearson correlation test. The significance of the correlation for each repeat and the variance of the RMSE over all repeats were considered. Furthermore, the mean and the standard error across all repeats was calculated for each case and summarized in a plot.

To extract the relevance of the different features, the frequency of the features selected for the model after mRMR and rfe within the 1000 computed models (10 folds, 100 repeats) was analysed. The modelling and statistical data analysis was implemented in R, version 4.2.1 [34] and SSPS, version 26.0 [35].

### Immunohistochemical analysis and semi-quantitative assessment of somatostatin receptors

Out of the 51 patients we identified 18 surgical specimens from histologically confirmed meningioma patients who had good tissue quality for immunohistochemical staining of SSTR. Please see **Supplement 2** for detailed description of the immunohistochemical analysis of somatostatin receptors (SSTR1, SSTR2A, SSTR2B, SSTR3, SSTR4, SSTR5). Semi-quantitative assessment of tissue receptor expression was performed using the immunoreactive-score (IRS). The IRS gives a range of 0–12 as a product of multiplication between staining intensity score (0 = no staining; 1 = 0.1–29%; 2 = 30–59.9%; 3 = 60–100%) and positive cells proportion score (0 = no positive cells, 1 = < 10% of positive cells, 10–50% positive cells, 51–80% positive cells, > 80% positive cells) [36]. SUV_max_ from [68Ga]Ga-DOTATOC PET/CT was calculated in these patients and Spearman rank test was used to correlate IRS with SUV_max_.

## Results

### Radiomic feature selection and prediction performance evaluation

**Fig. 1:**
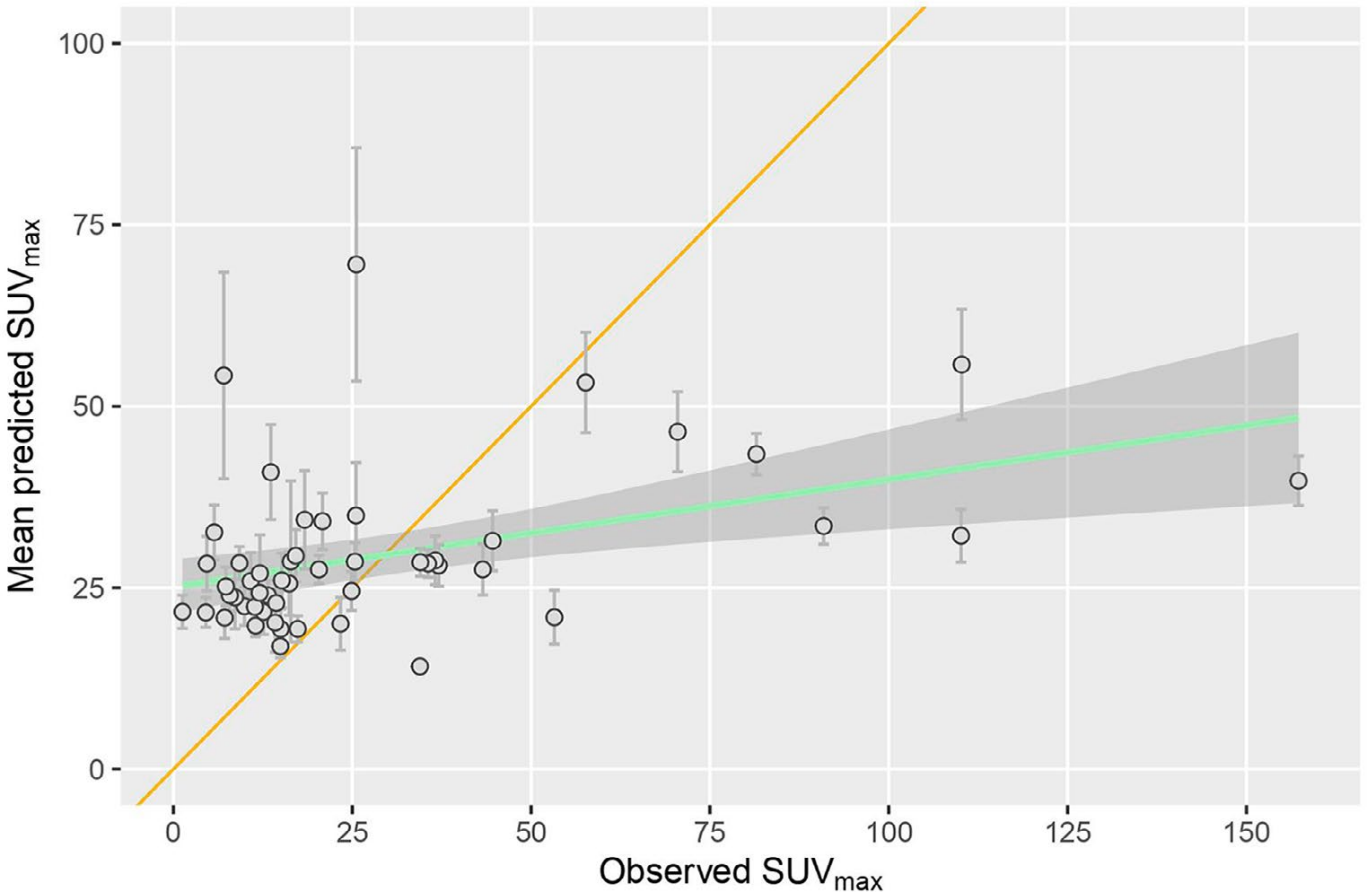
Mean SUV_max_ values predicted by the random forest models plotted against the observed SUV_max_ values for the 51 considered patients. The error bars indicate the standard deviation. The yellow line is the identity line. The green line and corresponding shaded area represents a linear regression fit to the data and its confidence interval

The SUV_max_ values predicted by the random forest regression models correlated significantly with the observed values (p < 0.05) for all 100 repeats with a mean Pearson’s r = 0.42 ± 0.07 SD and a RMSE = 28.46 ± 0.16. The mean predicted values plotted against the observed values are shown in Fig. 1.

**Fig. 2:**
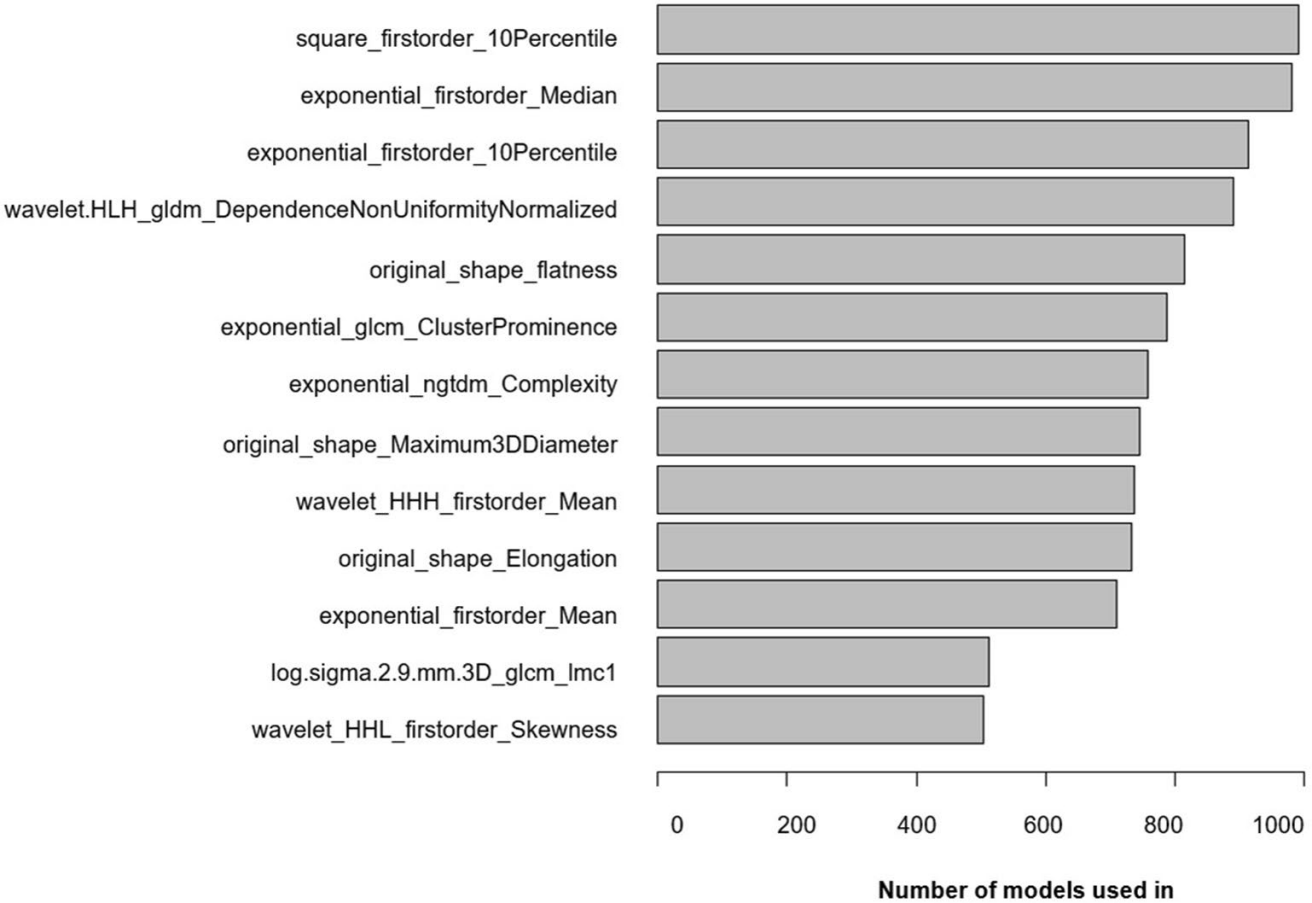
Radiomic features selected in more than 50% of all models

Across all folds and repeats, out of the 1940 unique radiomics features, a total of 220 different radiomic features were selected by the mRMR. The second step of feature reduction, the recursive feature elimination (rfe) within the random forest modelling, resulted in a feature sets of 2 to 30 features (median number of features is 25) for the prediction within each fold. The most dominant feature was selected as input data for 992 out of 1000 models. To give an overview on feature analysis results, the relative frequency of the features used in more than 500 out of 1000 models is shown in Fig. 2. Within this top ranked features, five different groups of radiomics can be identified: six first-order features, one gray-level dependence matrix (GLDM) feature, two gray-level co-occurrence matrix (GLCM) features, one neighboring gray-tone difference matrix (NGTDM) and three 3D Shape features (Table 2).

**Table 2:** Top ranked radiomic features categorised into five groups

First order	square_firstorder_10Percentile
	exponential_firstorder_Median
	exponential_firstorder_10Percentile
	wavelet.HHH_firstorder_Mean
	exponential_firstorder_Mean
	wavelet.HHL_firstorder_Skewness
GLDM	wavelet.HLH_gldm_DependenceNonUniformityNormalized
GLCM	exponential_glcm_ClusterProminence
	log.sigma.2.0.mm.3D_glcm_lmc1
NGTDM	exponential_ngtdm_Complexity
3D Shape features	original_shape_flatness
	original_shape_Maximum3DDiameter
	original_shape_Elongation

GLDM: Gray-level dependence matrix, GLCM: Gray-level cooccurrence matrix, NGTDM: neighboring gray-tone difference matrix

### Correlation between [68Ga]Ga-DOTATOC PET/CT and the SSTR expression intensity

Within the 18 tumour specimens, SSTR2A showed the highest immunoreactivity with a median IRS of 8, while SSTR1, SSTR2B und SSTR5 had a median IRS of 3 and SSTR3 und SSTR4 showed no immunoreactivity (median IRS = 0).

**Fig. 3:**
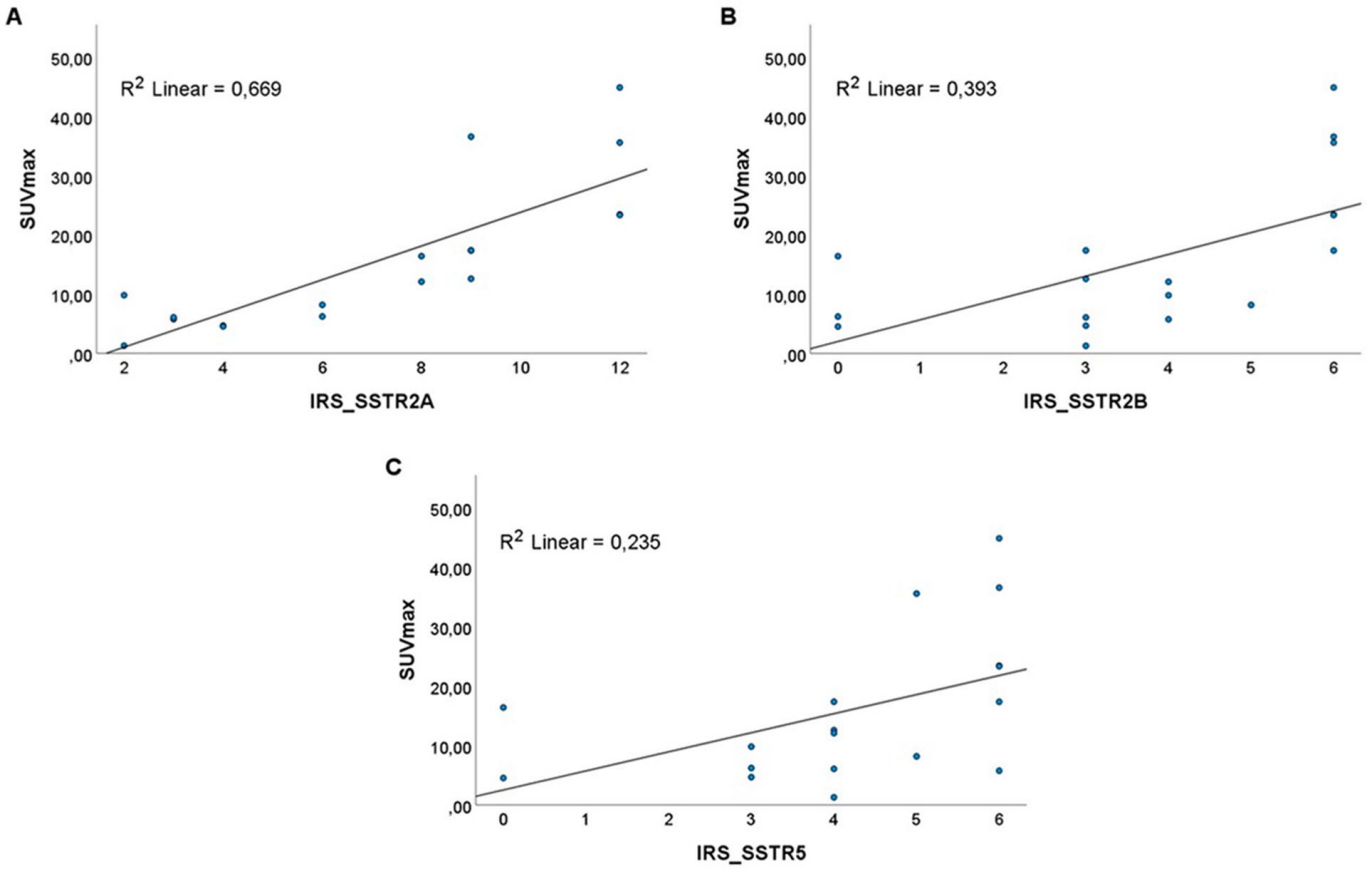
**A.** Significant correlation of IRS for SSTR2A with SUV_max_ (p < 0.001, R^2^ 0.669). **B.** Significant correlation of IRS for SSTR2B with SUV_max_ (p = 0.001, R^2^ 0.393). **C.** Significant correlation of IRS for SSTR5 with SUV_max_ (p = 0.012, R^2^ 0.235)

Analyses from preoperative [68Ga]Ga-DOTATOC PET/CT revealed a median SUV_max_ of 12,3 (range 1.3–44.9). The IRS for SSTR2A, SSTR2B and SSTR5 correlated significantly with the SUV_max_ on PET (p < 0.001, R^2^ = 0.669 for SSTR2A; p = 0.001, R^2^ = 0.393 for SSTR2B; p = 0.012, R^2^ = 0.235 for SSTR5) (Fig. 3).

## Discussion

In this retrospective study we immunohistochemically quantified SSTR subtypes in patients with resected meningioma and showed that SSTR subtypes 2A, 2B and 5 correlate significantly with SUV_max_ signal in [68Ga]Ga-DOTATOC PET/CT. In a second step we showed the potential of radiomic features derived from ADC maps from DWI MRI to model the [68Ga]Ga-DOTATOC PET/CT SUV_max_ signal in meningioma patients of different grades. The features with high explanatory value (selected in > 50% of all models) were dominated by first order, GLDM, GLCM, NGTDM and 3D Shape features.

In the first part of our study, we aimed to provide a pathophysiological background for SUV_max_ signal in [68Ga]Ga-DOTATOC PET. In PET, SUV came to be used as a tool to supplement visual interpretation and measures relative tissue uptake in comparison to other structures considering an optimal diagnostic threshold [37] thereby gaining additional information on tumour margins and tumour volume for possible radiotherapy or radionuclide therapy [13, 38].

So far, only one study [8] investigated the correlation between SSTR expression and SUV signal in [68Ga]Ga-DOTATOC PET in patients with meningioma. In 21 meningioma patients the authors found a significant positive correlation between SUV_max_ and SSTR2 expression and by analysing locally different biopsies a SUV_max_ cut off value of 2.3 was set to define tumorous tissue. A correlation subtype analysis in meningioma patients however has not been done so far. In our study we could confirm the correlation between SSTR2 and SUV_max_ signal. We furthermore could show that different subtypes correlate differently with SUV_max_ signal (SSTR2A correlated best followed by 2B and 5).

Only recently, a comprehensive analysis from 726 tumour samples showed a clear distinction of SSTR expression in meningioma subgroups. Especially, SSTR1, 2A, and 5 showed high expression rates [39]. The expression of SSTR2A has also shown to be an independent prognostic value regarding meningioma recurrence [40]. This finding is also important for further therapeutic consideration, as it relates to SSTR-targeted peptide receptor radionuclide therapy (PRRT), which represents a promising approach for treating refractory meningiomas that progress after surgery and radiotherapy [41, 42]. A deeper understanding on the distribution and role of somatostatin receptors in meningiomas is essential to further develop and refine a differentiated targeted application. PET with [68Ga]Ga-labelled somatostatin analogues has shown to assess the tumour radionuclide uptake in PRRT of meningioma prior to treatment and serves as an estimate of the achievable dose [38]. It has been demonstrated that a lesion-based analysis of SUV_max_ and SUV_mean_ in [68Ga]Ga-DOTATOC could predict response to PRRT [43] making [68Ga]Ga-DOTATOC PET an important predictive biomarker for PRRT. By showing that not only SSTR2 but especially SSTR2A, 2B and SSTR5 are highly correlated with SUV_max_ signal from [68Ga]Ga-DOTATOC we also provide more insight into the pathophysiology of the SUV_max_ signal and refine this pretherapeutically used diagnostic tool.

In the second part of the study, we established a predictive model to infer SUV_max_ values from radiomic features derived from ADC maps. Besides semantic or standard feature like tumour volume and signal intensity, radiomics has the ability to generate many more parameters that have been linked to specific tumour characteristics. In our study, 13 top ranked features which have been selected in the MRI model, were classified into five groups, as shown in Table 2. First-order statistics describe the distribution of voxel intensities within the VOI and showed to be a helpful tool in identifying brain invasion in meningiomas [44]. GLCM, GLDM and NGTDM are examples of textural features that are computed from gray level matrices extracted from a pre-segmented tumour. These features are then organized into groups based on the respective gray level matrices used in their extraction [45]. GLCM and GLDM provide valuable information on determining the optimal width for analysing invasiveness and peritumoural regions in meningioma [46]. GLCM features are utilized as biomarkers of heterogeneity, offering valuable insights into the tumour microenvironment [47]. In the case of meningiomas, NGTDM features, along with the other textural features, have demonstrated their usefulness in predicting Ki-67 and p53 status, as well as showing good performance in predicting progesterone receptor expression in high-grad meningiomas [48, 49]. Shape features, including 3D shape features, consist of descriptors that characterize the three-dimensional size and shape of ROI. These features are independent of the gray level intensity distribution of ROI. Several clinical trial have demonstrated that shape features extracted from MRI serve as informative imaging biomarkers for predicting high WHO grade and histological brain invasion in meningioma [50, 51].

To date, ADC radiomics in meningioma have only been investigated to predict meningioma grade [52] and outcome [53]. In a study of 71 meningioma patients, four statistically independent radiomic features derived from FLAIR, T1 contrast enhanced MRI and DWI MRI showed strong association with meningioma grades [52]. Using a decision forest classifier in 152 meningioma patients, built with 23 selected texture features and the ADC value an accuracy of 79.51% to predict meningioma grade was found [54]. Morin et al. [53] analysed prognostic models using clinical, radiologic (including ADC maps), and radiomic features to preoperatively identify meningiomas at risk for poor outcomes. Investigating 314 meningioma patients (57% WHO grade I, 35% grade II, and 8% grade III) at two independent institutions, they found that low ADC values were associated with high-grade meningioma, and low sphericity was associated with increased local failure and worse overall survival and the prediction of meningioma grading from preoperative brain MRI demonstrated good results in a meta-analysis [55].

Our results show that radiometric features derived from ADC maps can be significantly linked to the SUV_max_ signal. Therefore, our MR-based methodology could be of particular value for centers with limited access to PET imaging. Based on our findings, radiomics of ADC maps could be utilized in further studies to predict response to PRRT, similar to how it has been done by Park et al. in selecting radiotherapy for meningioma WHO grade II [56]. Certainly, as a limitation of this study, prospective studies are needed to show the full clinical utility of our model e.g. to detect tumour, for radiation planning, and to predict tumour growth. As a further benefit we state that diagnostic based on radiomics has the advantage of being reproducible. By now, depending on the physician doing the contouring of the tumour from lower resolution PET scans, the volume and the size of a meningioma can vary depending on the SUV threshold setting as there is no standardized procedure for selecting the intensity level [57]. This could be valuable for follow-up investigations.

In conclusion, in this study we could show that SSTR subtypes 2A, 2B and 5 correlate highly significantly with SUV_max_. We developed a radiomic model based on ADC maps derived from DWI MRI to model SUV_max_ from [68Ga]Ga-DOTATOC PET in meningiomas. Findings that may aid to increase the diagnostic as well as therapeutic accuracy in meningioma management.

### Electronic supplementary material

Below is the link to the electronic supplementary material.

Supplementary Material 1

Supplementary Material 2

## Data Availability

The datasets generated during and/or analysed during the current study are available from the corresponding author on reasonable request.

## Declarations

### Originality and Presentations

The authors confirm the originality of this study.
