# Supplementary material for: Somatostatin receptor subtype expression and radiomics from DWI-MRI represent SUV of [68Ga]Ga-DOTATOC PET in patients with meningioma
Source: J Neurooncol. 2023 Sep 14;164(3):711–20. doi: 10.1007/s11060-023-04414-3 (PMC10589159; doi:10.1007/s11060-023-04414-3)
Supplement: Supplementary file 1 — Supplementary Material 1 [file 11060_2023_4414_MOESM1_ESM.docx]

**Supplement Table 1.**

| Scanner Type | Number of subjects | Tesla | T1-weighted MRI | | | | DTI | | | | | |
| --- | --- | --- | --- | --- | --- | --- | --- | --- | --- | --- | --- | --- |
|  |  |  | TR | TE | Fa | Voxel size (mm) | TR | TE | Fa | Gradients | b-Values | Voxel size (mm) |
| Siemens Symphony Vision | 5 | 1.5 | 1710 | 4.38 | 15 | 0.9×0.9×5 | 6100 | 108 | 90 | 3 | 0, 1000 | 0.9×0.9×5 |
| Siemens Symphony Tim | 26 | 1.5 | 1830 | 3,62 | 15 | 0.9×0.9×1.2 | 3000 | 80 | 90 | 12 | 0, 1000 | 1.8×1.8×5 |
| Siemens Avanto | 1 | 1.5 | 1600 | 3.44 | 15 | 0.9×0.9×1.2 | 6000 | 94 | 90 | 12 | 0, 1000 | 0.9×0.9×3 |
| Siemens Sonata | 1 | 1.5 | 564 | 14 | 90 | 0.7×0.7×5 | 3700 | 107 | 90 | 3 | 0, 1000 | 1.8×1.8×5 |
| Siemens Skyra | 9 | 3 | 1820 | 2.96 | 8 | 0.8×0.8×1 | 7000 | 95 | 90 | 20 | 0, 1000 | 0.9×0.9×3 |
| GE optima | 1 | 1.5 | 621 | 1.96 | 12 | 0.8×0.8×1 | 8571 | 106 | 90 | 6 | 0, 1000 | 0.8×0.8×4 |
| Philips Achieva | 3 | 1.5 | 500 | 12 | 70 | 0.7×0.7×5 | 4600 | 80 | 90 | 3 | 0, 1000 | 0.9x0.9x5 |
| Siemens Aera | 5 | 1.5 | 1870 | 3.33 | 8 | 0.9×0.9×1 | 7000 | 97 | 90 | 20 | 0, 1000 | 1.7x1.7x3 |
