# Supplementary material for: Somatostatin receptor subtype expression and radiomics from DWI-MRI represent SUV of [68Ga]Ga-DOTATOC PET in patients with meningioma
Source: J Neurooncol. 2023 Sep 14;164(3):711–20. doi: 10.1007/s11060-023-04414-3 (PMC10589159; doi:10.1007/s11060-023-04414-3)
Supplement: Supplementary file 2 — Supplementary Material 2 [file 11060_2023_4414_MOESM2_ESM.docx]

**Supplement 2**

**Immunohistochemical analysis and semi-quantitative assessment of somatostatin receptors**

Two micrometer thick paraffin sections were used and sections were dewaxed followed by antigen retrieval. Rabbit polyclonal primary antibodies (Abcam, Cambridge, UK) were added to the section by manual titration and staining was completed by using a Discovery automated staining system as well as haematoxylin counterstaining and diamino-benzamidine Map Kit (Ventana, Tucson, Arizona, USA). The immunohistochemical staining reaction was confirmed regarding to pituitary specimen, which served as positive control, and tissue from basal ganglia provided as negative control. Subsequently, two blinded observers evaluated independently the immunhistochemical reaction in tumor cell nests, which have been collected from ten areas from each specimen. These areas were analysed by using an Olympus BX50 light microscope (Tokyo, Japan). (36). Subsequently, the staining reaction was converted to a staining intensity score.
